# Philadelphia Chromosome-Positive De Novo Acute Myeloid Leukemia Treated With Chemotherapy and Second-Generation Tyrosine Kinase Inhibitor

**DOI:** 10.7759/cureus.5135

**Published:** 2019-07-15

**Authors:** Juan D Muñoz, Gilberto Israel Barranco Lampón, Humberto Castellanos, Christian Ramos, Juan F Zazueta

**Affiliations:** 1 Hematology, Hospital General De México, Mexico City, MEX; 2 Hematology, Hopsital General De México, Mexico City, MEX; 3 Hematología, Hospital General De México, Mexico City, MEX

**Keywords:** acute myeloid leukemia, philadelphia chromosome, tyrosine kinase inhibitor

## Abstract

Philadelphia chromosome-positive Acute Myeloid Leukemia (AML) is a de novo acute leukemia in which patients show no evidence of Chronic Myeloid Leukemia (CML) before or after their treatment. This kind of leukemia has an aggressive clinical course, with poor response to traditional chemotherapy or monotherapy with Tyrosine Kinase Inhibitors (TKI), and a high risk of early relapse after induction therapy. We report a rare case of de novo ALM with t(9;22). A 26-year-old male patient was referred to our hospital for an examination of anemia, thrombocytopenia (hemoglobin 5.7 g/dL and platelets 110 000/L) and elevated White Blood Cell (WBC) count (11 600 μ/L, 24% segmented, 63% lymphocytes, 11% monocytes). Bone marrow smear was compatible with AML. Cytogenetic study revealed t(9;22)(q34;q11). Our patient was treated with chemotherapy for AML and a second-generation TKI and remains in complete remission pending a bone marrow transplant.

## Introduction

Acute Myeloid Leukemia (AML) with BCR-ABL (an aberrant tyrosine kinase resulting from a fusion protein product of the acquired Philadelphia chromosome) corresponds to a provisional entity of the 2016 revised World Health Organization (WHO) classification of tumors of the hematopoietic and lymphoid tissues, which defines it as a de novo AML in which patients show no evidence of Chronic Myeloid Leukemia (CML) before or after their treatment. Excluded from this category are mixed phenotype leukemias, myeloid neoplasms associated with treatment, and AML with recurrent genetic abnormalities. AML with BCR-ABL corresponds to <1% of all AML and <1% of all acute and chronic positive BCR-ABL leukemias. It occurs predominantly in male adult patients. Patients with Philadelphia Chromosome-Positive De Novo AML have an aggressive clinical course, with poor response to traditional chemotherapy or monotherapy with Tyrosine Kinase Inhibitors (TKI) and a high risk of early relapse after induction therapy [1-4].

## Case presentation

**Figure 1:**
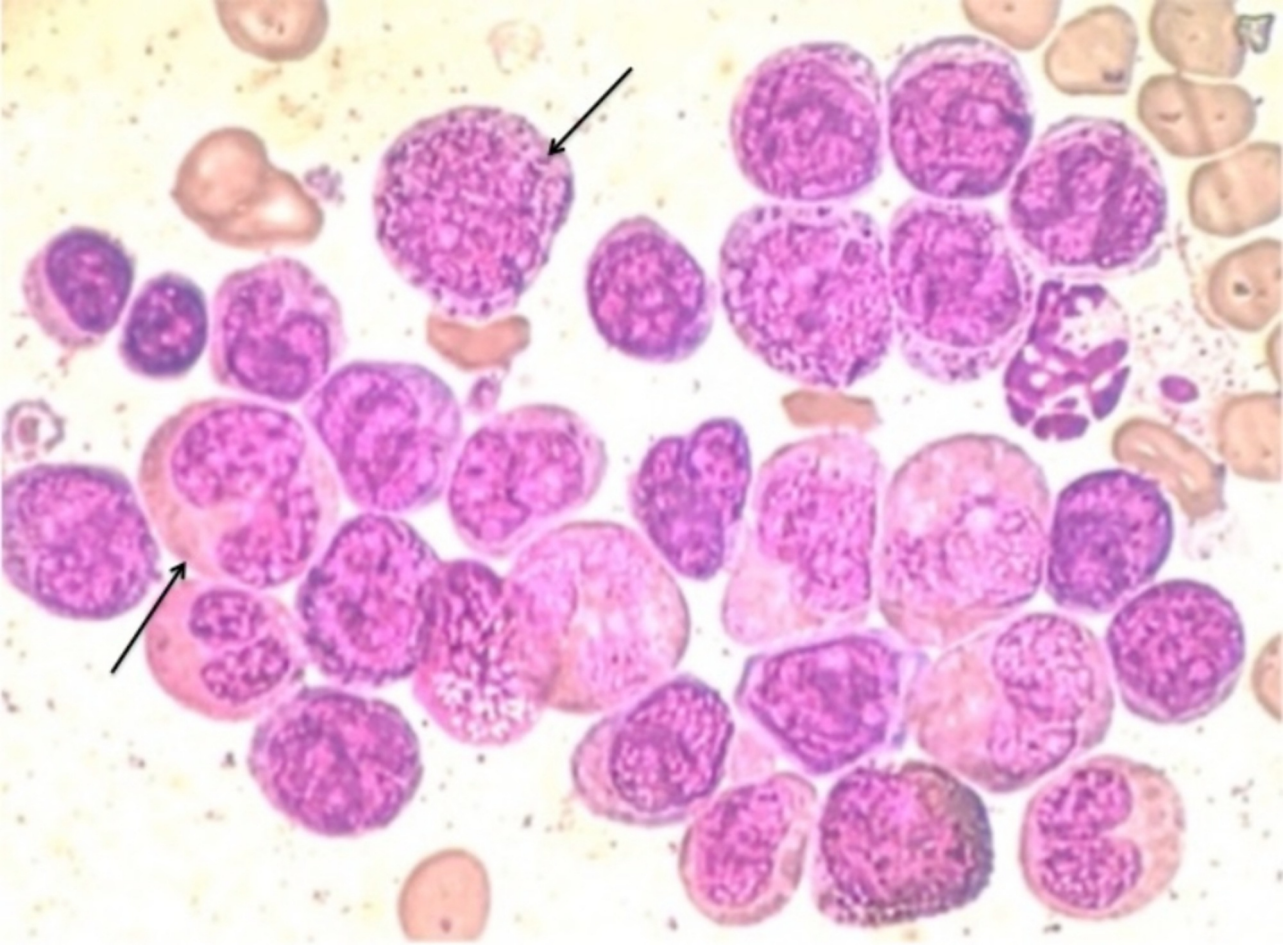
Bone marrow aspirate Bone marrow aspirate showing infiltration by myelomonoblastic cells and a slight increase of monocytes with severe erythroid, granulocytic and lymphocyte depression.

A 26-year-old male patient from Mexico City presented to the Emergency Department with a history of asthenia, adynamia, generalized weakness, nausea, dizziness, and diarrhea. He had no occupational exposure and no associated comorbidity was present. Physical examination revealed ecchymosis in upper limbs. His laboratory data on admission showed elevated White Blood Cell (WBC) count (11600/μL, 24% segmented, 63% lymphocytes, 11% monocytes) with anemia and thrombocytopenia (hemoglobin 5.7 g/dL and platelets 110000/μL). Coagulation studies were normal. His bone marrow aspirate revealed a hypocellular specimen with 90% blasts, absent megakaryocytes, significant infiltration by myelomonoblastic cells and a slight increase of monocytes with severe erythroid, granulocytic and lymphocyte depression (Figure 1). Morphological findings were compatible with AML.

**Figure 2:**
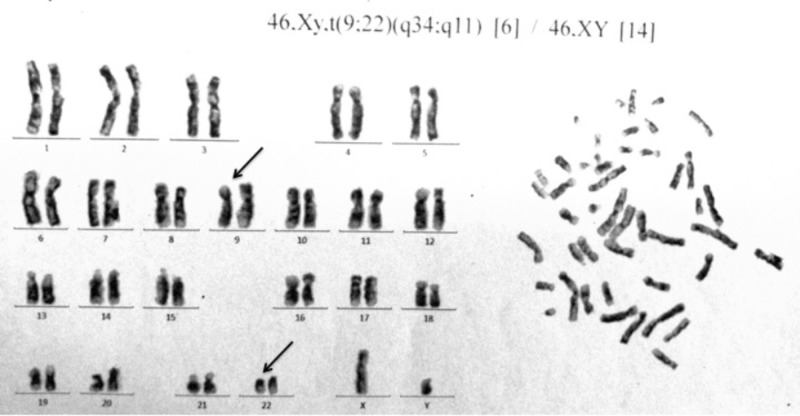
G-banded karyotype of the bone marrow cells showing t(9;22)(q34;q11).

Immunophenotype with multicolor flow cytometry showed positivity (>30%) for Cluster of Differentiation (CD) 34 (70%), CD117 (70%), CD13 (66%), CD33 (99%), CD64 (49%), myeloperoxidase (MPO) was negative (23%). Chromosomal analysis with G-banded karyotype of the bone marrow cells showed 46, XY, t(9;22)(q34;q11) in 6 of all 20 metaphase spreads (Figure 2). BCR-ABL qualitative diagnostic assay was positive.

 

The patient was treated according to the institutional induction protocol with daunorubicin (60mg/m2) and cytarabine (100mg/m2) for three and seven days, respectively. The bone marrow aspirate at the end of the induction phase revealed complete remission (CR). Subsequently, the patient received consolidation therapy with cytarabine (2g/m2 twice a day, days 1,3 and 5) every 28 days for three cycles. Nilotinib was prescribed during induction, consolidation and as maintenance at a dose of 400 mg twice daily. Currently, 15 months after diagnosis, the patient remains in remission awaiting allogeneic bone marrow transplantation from a Human Leukocyte Antigen (HLA)-compatible donor.

## Discussion

AML with BCR-ABL corresponds to a provisional entity of the classification of tumors of the hematopoietic and lymphoid tissues of the WHO in its fourth revised edition. It occurs predominantly in adults of the male gender [1-3].

Patients often present with leukocytosis, varying degrees of anemia, and thrombocytopenia. Unlike patients with CML in myeloid blast phase, they have a lower degree of splenomegaly and fewer basophils in the blood (<2%) [2]. The morphological characteristics are not specific; there are myeloid blasts in peripheral blood and bone marrow with minimal granulocytic maturation. The average cellularity of the bone marrow and the number of dwarf megakaryocytes are lower than those found in the myeloid blast transformation of the CML (cellularity: 80% vs 95-100%). The ratio of non-blast myeloid: erythroid cells is normal compared to that of CML in myeloid blast phase [1-3].

There are few studies that address the immunophenotype of this disease; however, what is documented is positivity for myeloid antigens (CD13 and CD33) and CD34. Frequently aberrant expression of CD19, CD7 and Terminal deoxynucleotidyl transferase (TdT) are present. The normal counterpart that has been postulated corresponds to a hematopoietic progenitor cell with multilineal potential [4]. Unlike the cases with acute leukemia with mixed phenotype and t (9; 22) (q34.1; q11.2) in which the immature cell population can express lineage B or T antigens, our case expressed only myeloid antigens.

In relation to the genetic profile, all cases exhibit t(9; 22) (q34.1; q11.2) or molecular evidence of the BCR-ABL fusion gene. Most patients have a fusion of molecular weight p210 (with cleavages b2a2 and b3a2 as more common). In a few cases, the minor breakage has been documented (p190). Other common genetic abnormalities are the loss of chromosome 7, a gain of chromosome 8, as well as complex karyotypes [1-4].

In addition, mutations of Nucleophosmin (NPM-1) and FMS like tyrosine kinase 3 with internal tandem duplication (FLT3-ITD), loss of Ikaros (IKZF1) and cyclin-dependent kinase Inhibitor 2A (CDKN2A) and cryptic deletions within the genes of Immunoglobulin Heavy Chain (IGH) and T cell receptor gamma locus (TRG) have been reported (infrequently). All these alterations are not found in CML in the blast phase. The genetic alterations that define AML with recurrent genetic abnormalities should exclude the diagnosis of this pathology [4-8]. It should be noted that the late acquisition of BCR-ABL has been reported in patients with AML, but it also does not define this pathological entity [5-9].

This disease appears to be aggressive with poor response to traditional therapy or inhibition of tyrosine kinase if they are tried in isolation [5]. Recent studies document improvement in survival with inhibitors of tyrosine kinase and subsequent allogeneic transplantation of hematopoietic stem cells [6-9].

On the basis of the morphology, the immunophenotype of the blasts, and the molecular and cytogenetic analysis at diagnosis we conclude that this was a case of de novo Philadelphia chromosome-positive AML, in order to obtain a better response to conventional treatment with chemotherapy we add a second generation TKI. The patient remains in complete remission and due to the risk of the disease, an allogeneic transplant will be performed.

## Conclusions

Identifying chromosomal abnormalities associated with hematological malignancies is essential because they are important parameters for diagnosis, determination of prognosis and treatment modalities. Thanks to the identification of t (9; 22) we were able to add a TKI to conventional treatment and the clinical course of this patient is progressing satisfactorily.

## References

[1] Arber DA, Orazi A, Hasserjian R (2016). The 2016 revision to the World Health Organization classification of myeloid neoplasms and acute leukemia. Blood.

[2] Konoplev S, Yin CC, Kornblau SM (2013). Molecular characterization of de novomo Philadelphia chromosome-positive acute myeloid leukemia. Leuk Lymphoma.

[3] Paietta E, Recevkis J, Bennett JM (1998). Biologic heterogeneity in Philadelphia chromosome-positive acute leukemia with myeloid morphology: the Eastern Cooperative Oncology Group experience. Leukemia.

[4] Soupir CP, Vergilio JA, Dal Cin P (2007). Philadelphia chromosome-positive acute myeloid leukemia: a rare aggressive leukemia with clinicopathologic features distinct from chronic myeloid leukemia in myeloid blast crisis. Am J Pathol.

[5] Han JY, Theil KS (2006). The Philadelphia chromosome as a secondary abnormality in inv(3)(q21q26) acute myeloid leukemia at diagnosis: confirmation of p190 BCR-ABL mRNA by real-time quantitative poliymerase chain reaction. Cancer Genet Cytogenet.

[6] Roth CG, Contis L, Gupta S, Agha M, Safyan E (2011). De novo acute myeloid leukemia with Philadelphia chromosome (BCR-ABL) and inversion 16 (CBFB-MYH11): report of two cases and review the literature. Leuk Lymphoma.

[7] Quintás-Cardama A, Gibbons DL, Cortés J (2008). Association of 3q21q26 syndrome and late-appearing Philadelphia chromosome in acute myeloid leukemia. Leukemia.

[8] Stah N, Leaker MT, Teshima I, Baruchel S, Abdelhaleem M, Ye CC (2008). Late-appearing Philadelphia chromosome in childhood acute myeloid leukemia. Pediatr Blood cancer.

[9] Bhatt VR, Akhtari M, Bociek RG (2014). Allogenic stem cell transplantation for Philadelphia chromosome-positive acute myeloid leukemia. J Natl Compr Canc Netw.

